# Perinatal outcomes among immigrant mothers over two periods in a region of central Italy

**DOI:** 10.1186/1471-2458-11-294

**Published:** 2011-05-10

**Authors:** Laura Cacciani, Simona Asole, Arianna Polo, Francesco Franco, Renato Lucchini, Mario De Curtis, Domenico Di Lallo, Gabriella Guasticchi

**Affiliations:** 1 Laziosanità - Agency for Public Health of Lazio Region, Via di Santa Costanza, 53, 00198 - Rome, Italy; 2 Department of Paediatrics, La Sapienza University of Rome, Rome, Italy

## Abstract

**Background:**

The number of immigrants has increased in Italy in the last twenty years (7.2% of the Italian population), as have infants of foreign-born parents, but scanty evidence on perinatal outcomes is available. The aim of this study was to investigate whether infants of foreign-born mothers living in Italy have different odds of adverse perinatal outcomes compared to those of native-born mothers, and if such measures changed over two periods.

**Methods:**

The source of this area-based study was the regional hospital discharge database that records perinatal information on all births in the Lazio region. We analysed 296,739 singleton births born between 1996-1998 and 2006-2008. The exposure variable was the mother's region of birth. We considered five outcomes of perinatal health. We estimated crude and adjusted odds ratios and 95% confidence intervals (CIs) to evaluate the association between mother's region of birth and perinatal outcomes.

**Results:**

Perinatal outcomes were worse among infants of immigrant compared to Italian mothers, especially for sub-Saharan and west Africans, with the following crude ORs (in 1996-1998 and 2006-2008 respectively): 1.80 (95%CI:1.44-2.28) and 1.95 (95%CI:1.72-2.21) for very preterm births, and 1.32 (95%CI:1.16-1.50) and 1.32 (95%CI:1.25-1.39) for preterm births; 1.18 (95%CI:0.99-1.40) and 1.17 (95%CI:1.03-1.34) for a low Apgar score; 1.22 (95%CI:1.15-1.31) and 1.24 (95%CI:1.17-1.32) for the presence of respiratory diseases; 1.47 (95%CI:1.30-1.66) and 1.45 (95%CI:1.34-1.57) for the need for special or intensive neonatal care/in-hospital deaths; and 1.03 (95%CI:0.93-1.15) and 1.07 (95%CI:1.00-1.15) for congenital malformations. Overall, time did not affect the odds of outcomes differently between immigrant and Italian mothers and most outcomes improved over time among all infants. None of the risk factors considered confounded the associations.

**Conclusion:**

Our findings suggest that migrant status is a risk factor for adverse perinatal health. Moreover, they suggest that perinatal outcomes improved over time in some immigrant women. This could be due to a general improvement in immigrants' health in the past decade, or it may indicate successful application of policies that increase accessibility to mother-child health services during the periconception and prenatal periods for legal and illegal immigrant women in Italy.

## Background

Immigration in Italy is an important and widespread phenomenon. The number of immigrants living in the country has progressively increased in the last twenty years, from 650,000 at the beginning of the 90's [1] to 4,330,000 in 2008, representing 7.2% of the Italian population [2].

Immigration was initially and primarily characterized by young male adults looking for work. Recently, the number of women has increased, particularly those from Eastern Europe, due to more job opportunities in Italy compared to in their countries of origin, and to family re-unification [2,3]. Following the stabilization of immigrant families, and enlargement of the European Union to include Eastern European countries in 2004, the number of infants of foreign born parents has also increased, reaching about 72,500 newborns in 2008, 12.6% of all newborns in the nation [2].

In 2008, 10% of immigrants were resident in Lazio, a region of central Italy. Demographic characteristics of the foreign population in this region are similar to those observed at the national level. The main reasons for immigrating to Lazio were employment (57%) and family reunification (26%); about 2% immigrated for humanitarian and asylum reasons [4].

Perinatal outcomes have been extensively studied among immigrant women in different countries, although evidence is scarce in Italy [5-7]. The international literature shows conflicting results on this issue [8-10].

Some studies have indicated immigrant status as a risk factor [11], showing a higher rate of prematurity [6,12-14], lower birth weight [13] and a low Apgar score at 5 minutes [14,15], a higher proportion of children with neonatal asphyxia [13] and perinatal mortality [13,16] among immigrant compared to native-born populations. Some studies have shown higher percentages of congenital malformation among infants born to migrant women [7,17-19]. Possible explanations argued by the authors were poor prenatal care for migrants compared to Italians, or unfavourable socio-economic factors.

Other studies found that being an immigrant was a protective factor for perinatal outcomes. The risk of preterm delivery was lower among infants born to Somali mothers in four European countries and in Australia and Canada [20]; among Mexican women in Chicago [21]; and among immigrants in the US and Belgium [22]. Infants born to immigrant women in the US, Belgium, and France had lower risks of low birth weight compared to the native-born population [22,23]. In a Spanish study immigrant infants showed lower risks of low birth weight and prematurity compared to those of native-born women [24]. Such contrasting evidence may be explained by different access and referral to healthcare services [8,13,25], and by different integration policies in the host countries [11]. Better prognoses have been often explained also by the healthy migrant effect [23,26,27] and the epidemiological paradox - i.e. better perinatal outcomes among foreign-born women with demographic and socio-economic risk factors [27-29].

Known risk factors of adverse perinatal outcomes include: advanced maternal age [30-32], maternal psychosocial stress and poor education [33-35], low neighbourhood income and deprivation [35-37], nulliparity [38], and maternal unmarried status [39,40]. Among immigrants, length of stay in the host country has been also considered a risk factor that resulted associated with increases in the risk of preterm delivery [40,41].

The Italian health system provides universal coverage for hospital care. Legal immigrants are entitled to utilize the National Health Service. A variety of essential services are offered free of charge to all, including to illegal immigrants (i.e. emergencies, maternal and child clinics, mandatory vaccinations, hospital and ambulatory care for conditions which could represent severe long-term health problems if left untreated) [42]. A national protocol regulated by a Decree of the Ministry of Health in 1998 established a number of free prenatal screening tests (ultrasound or serology), including amniocentesis for women over 35 years old or those at risk for congenital anomalies. However, cultural and linguistic barriers may limit the access to health services during pregnancy, and the lack of cultural mediators in prenatal clinics may lead to inequalities in pregnancy surveillance, especially for newcomers and illegal immigrants.

Independent of their country of origin, immigrants can be granted Italian citizenship in specific circumstances (having an Italian relative, marriage to an Italian, or after legal residence for at least ten years).

The aim of this study was to investigate whether infants of foreign-born mothers living in Lazio had different odds of adverse perinatal outcomes compared to those of Italian-born mothers, and whether such measures changed over two periods in which immigrant births increased and legislative measures improved accessibility to prenatal care.

## Methods

**Figure 1:**
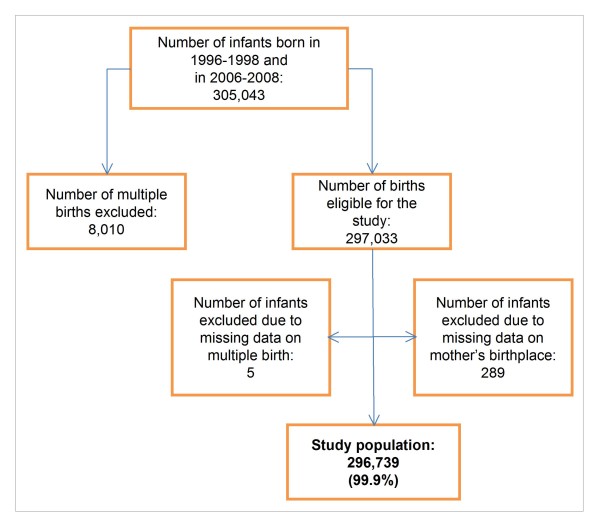
Flow-chart of the study population

The study referred to the Lazio region (5,6 million inhabitants), one of the 20 administrative Italian regions, and includes the capital, Rome. The data were retrieved only from the hospital discharge database that records selected perinatal information for all live births, including principal and secondary diagnoses (e.g. on birth defects). We selected 297,033 singleton live births out of 305,043 that occurred over two three-year periods (1996-1998 and 2006-2008) and excluded 294 infants (<0.1%) because of missing information regarding multiple birth status or the mother's country of origin, leaving 296,739 births in the final study population (figure 1).

The exposure variable was immigrant status as identified by the mother's country of birth. We subdivided the study population into infants born to Italians, and infants born to mothers from seven different world regions according to geographic, ethnic, or migration flow similarities: Western developed countries (DCs), Eastern Europe, North Africa, West and sub-Saharan Africa, Central and South America, Western and Central-Southern Asia, East Asia (see Additional file 1).

We considered five outcome variables: gestational age (GA, defined as the best obstetrical estimate): ≤31 weeks (very preterm births, VPBs), 32-36 (preterm births, PBs), and ≥37 weeks (full term); Apgar score at 5 minutes: 0-6 (low), and 7-10 (normal); principal or secondary diagnosis in the hospital discharge records (ICD9CM codes) of neonatal respiratory disorders, as the presence of either neonatal asphyxia (768) or respiratory diseases (769-770); need for special or intensive neonatal care, defined as transfers from delivery room to neonatal unit within or outside the facility, and in-hospital deaths, considered as a proxy of intensive care; principal or secondary diagnosis of congenital malformations (ICD9CM codes: 740-759).

Potential confounders considered were: maternal age groups (15-19, 20-24, 25-34, 35-44, 45+ years); self-reported educational level attained (none or elementary school, middle school, high school, higher education) and marital status (unmarried, married, separated, divorced, widowed) available only in 2006-2008; gestational week at first visit (<12, 12+); parity (0, 1, 2+ previous newborns). We considered the periods of time (1996-1998; 2006-2008) and Italian citizenship (available only in 2006-2008) as additional factors associated with the outcomes, and we assumed that they could interact with exposure. The choice of the above-mentioned covariates was primarily based on the existing literature.

### Statistical analysis

Descriptive statistics and frequency distributions of exposure and outcomes were calculated with chi-squared and Wilcoxon rank sum tests.

Crude and adjusted odds ratios (ORs) for all the above-mentioned confounders (using a forward-fitting strategy) were estimated by multivariate logistic regression to evaluate the association between mother's place of birth and neonatal outcomes (the polytomous approach was run for GA, using the mlogit command that estimates a relative risk ratio resembling the odds ratio given by the logistic command), stratifying by time period (model 1a for 1996-1998 and model 1b for 2006-2008) and mother's region of birth. Only crude measures were reported in the final model because the selected adjusting variables did not show any confounding effect on the associations. Furthermore, crude ORs comparing the second period with the first period within each world region were calculated (model 2) in order to test changes of outcomes over time. Robust standard errors and robust 95% confidence intervals (95%CIs) were calculated to take into account the cluster effect of the mother's birthplace in all the models.

Interactions of time and citizenship were assessed by a stratified analysis (results not shown).

The Wald test was used to identify ORs that were different from one. Results were considered statistically significant when p-values (two-tailed) were less than 0.05. Percentages of missing data were always less than 1% except for GA (5%) among Eastern Europeans. StataSE 11 software was used for the statistical analysis.

Ethics committee approval is not required for observational studies that use anonymous administrative data, where human subjects do not receive any treatment; results are shown in aggregate form and identification of individuals is not possible.

## Results

Overall, 296,739 newborns were analysed. There were 14,901 infants born to immigrant mothers in the first period (11%) and 33,830 in the second (21%) [43].

In the first period, the most represented countries were the Philippines (9.4%) followed by Eastern European countries, while in the second, the majority of immigrants came from Romania (35%); births reflected the change in population flows (table 1).

**Table 1:** Region and country of birth of immigrant mothers. Lazio, 1996-1998 and 2006-2008

Mother's region or country of birth*	Years 1996-1998			Mother's region or country of birth*	Years 2006-2008	
	n	%			n	%
Western developed countries	3884	26.1		Western developed countries	3683	10.9
*France*	*615*	*4.1*		*Germany*	*679*	*2.0*
*Switzerland*	*553*	*3.7*		*Switzerland*	*545*	*1.6*
*United Kingdom*	*470*	*3.2*		*France*	*497*	*1.5*
Eastern Europe	3781	25.4		Eastern Europe	18650	55.1
*Poland*	*1273*	*8.5*		*Romania*	*11841*	*35.0*
*Montenegro*	*753*	*5.1*		*Poland*	*1859*	*5.5*
*Romania*	*641*	*4.3*		*Albania*	*1442*	*4.3*
North Africa	1310	8.8		North Africa	1889	5.6
*Egypt*	*373*	*2.5*		*Morocco*	*753*	*2.2*
*Morocco*	*358*	*2.4*		*Egypt*	*555*	*1.6*
*Tunisia*	*309*	*2.1*		*Tunisia*	*338*	*1.0*
West and sub-Saharan Africa	1064	7.1		West and sub-Saharan Africa	1469	4.3
*Ethiopia*	*229*	*1.5*		*Nigeria*	*403*	*1.2*
*Nigeria*	*209*	*1.4*		*Ethiopia*	*278*	*0.8*
*Cape Verde*	*135*	*0.9*		*Cape Verde*	*90*	*0.3*
Central and South America	2030	13.6		Central and South America	3588	10.6
*Peru*	*558*	*3.7*		*Peru*	*905*	*2.7*
*Brazil*	*370*	*2.5*		*Ecuador*	*573*	*1.7*
*Venezuela*	*244*	*1.6*		*Brazil*	*553*	*1.6*
Western and Central-Southern Asia	916	6.1		Western and Central-Southern Asia	2061	6.1
*India*	*360*	*2.4*		*Bangladesh*	*737*	*2.2*
*Sri Lanka (Ceylon)*	*190*	*1.3*		*India*	*598*	*1.8*
*Bangladesh*	*144*	*1.0*		*Sri Lanka (Ceylon)*	*371*	*1.1*
East Asia	1916	*12.9*		East Asia	*2490*	*7.4*
*Philippines*	*1404*	*9.4*		*Philippines*	*1324*	*3.9*
*China*	*349*	*2.3*		*China*	*1027*	*3.0*
*South Korea*	*64*	*0.4*		*Thailand*	*45*	*0.1*
Total	14901	100.0		Total	33830	100.0

* Only the three most frequent countries within each region are shown.

The percentage of Italian citizens ranged from 63% (DCs) to 17.0% (Eastern Europeans).

Table 2 shows the distribution of selected maternal characteristics by mother's region of birth and time period. On average, foreign mothers were younger than Italians, in particular Eastern Europeans (6.6% aged <20 years). Age at delivery increased among Italians (median 30 to 33; p < 0.0001), but decreased among immigrants (from 29 to 28; p < 0.02). Low educational level was more common in immigrants than in Italians (17% vs. 11%), with the most educated coming from DCs (26%) and the less educated from Africa and Asia (about 60% had only a middle school diploma). The highest percentage of unmarried mothers was from Central and South America (61%). Although immigrant mothers compared to Italians showed higher percentages of a late first prenatal visit (especially East Asians), these decreased from 13% to 6.5% (p < 0.0001). Multiparity was highest among mothers from North Africa and the lowest among Italians (23% vs.9.3%).

**Table 2:** Selected maternal characteristics and perinatal outcomes by mother's region of birth. Lazio, 1996-1998 and 2006-2008

	Italy		Western developed countries		Eastern Europe		North Africa	
	'96-'98	'06-'08	'96-'98	'06-'08	'96-'98	'06-'08	'96-'98	'06-'08
	N = 120886	N = 127122	N = 3884	N = 3683	N = 3781	N = 18650	N = 1310	N = 1889
MATERNAL CHARACTERISTICS								

Mother's age*								

15-19	1.0	1.0	0.5	0.3	6.6	5.9	2.3	1.7

20-24	9.1	5.5	8.2	3.0	30.0	23.7	16.0	16.5

25-34	70.4	57.9	71.5	52.9	55.3	58.0	58.9	56.7

35-44	19.3	35.3	19.5	43.5	7.7	12.2	22.4	24.7

45-59	0.2	0.3	0.2	0.3	0.2	0.1	0.2	0.3

Missing	0.1	0.0	0.1	0.0	0.2	0.1	0.2	0.0

Educational level attained*								

None or elementary school	-	10.6	-	14.2	-	15.2	-	21.2

Middle school	-	25.2	-	18.4	-	41.4	-	41.5

High school	-	47.1	-	41.6	-	37.2	-	28.0

Higher education	-	17.0	-	25.7	-	6.0	-	9.3

Missing	-	0.2	-	0.2	-	0.2	-	0.1

Marital status*								

Unmarried	-	25.1	-	24.4	-	29.7	-	9.2

Married	-	71.8	-	72.2	-	66.9	-	88.6

Separated	-	1.6	-	1.3	-	0.9	-	0.7

Divorced	-	0.8	-	1.2	-	1.3	-	0.3

Widowed	-	0.1	-	0.1	-	0.1	-	0.0

Missing	-	0.6	-	0.7	-	1.1	-	1.2

First prenatal visit (weeks)*								

<12	94.8	98.8	94.5	98.6	84.1	92.8	90.2	93.8

≥12	5.2	1.2	5.5	1.3	15.8	6.9	9.8	5.9

Missing	0.0	0.0	0.0	0.1	0.1	0.3	0.0	0.3

Parity*								

0	53.1	56.3	51.4	53.7	59.9	61.1	44.7	40.1

1	37.0	35.1	37.5	35.3	24.6	28.2	37.1	33.9

≥2	10.0	8.6	11.0	11.0	15.5	10.7	18.2	26.0

PERINATAL OUTCOMES								
Gestational age (weeks)								

<31	0.7	0.6	0.8	0.5	1.1	1.3	0.5	0.6

32-36	4.8	5.2	4.9	5.1	6.1	6.7	6.9	5.1

37+	93.8	94.1	93.3	94.4	88.0	92.0	91.5	94.3

Missing	0.7	0.0	1.0	0.0	4.8	0.0	1.1	0.0

Five Minute Apgar Score								

0-6	1.7	1.4	2.0	1.1	1.9	1.4	2.1	1.8

7-10	97.8	98.6	97.0	98.9	97.5	98.6	97.8	98.2

Missing	0.4	0.0	1.0	0.0	0.5	0.0	0.2	0.0

Respiratory diseases	6.9	4.6	7.3	3.7	8.0	5.9	8.9	6.4

Special/intensive care	3.8	3.6	3.4	3.0	5.1	5.2	4.8	4.6

Congenital Malformations	3.8	5.4	3.2	4.9	4.2	5.6	3.2	6.0

	West and sub-Saharan Africa		Central and South America		Western and Central-Southern Asia		East Asia	
	'96-'98	'06-'08	'96-'98	'06-'08	'96-'98	'06-'08	'96-'98	'06-'08
	N = 1064	N = 1469	N = 2030	N = 3588	N = 916	N = 2061	N = 1916	N = 2490

MATERNAL CHARACTERISTICS								

Mother's age*								

15-19	0.9	1.6	2.0	2.7	1.1	1.3	0.8	2.2

20-24	12.6	9.3	13.8	12.4	23.0	19.7	11.8	16.1

25-34	64.6	60.9	60.0	57.1	61.0	64.5	66.3	56.3

35-44	21.4	27.9	23.9	27.5	14.4	14.1	20.8	24.8

45-59	0.4	0.3	0.1	0.3	0.4	0.3	0.2	0.6

Missing	0.1	0.0	0.1	0.0	0.0	0.0	0.1	0.0

Educational level attained*								

None or elementary school	-	21.4	-	15.2	-	23.6	-	22.3

Middle school	-	40.0	-	33.7	-	43.5	-	39.6

High school	-	32.4	-	40.8	-	25.6	-	31.2

Higher education	-	6.1	-	9.9	-	7.0	-	6.8

Missing	-	0.1	-	0.3	-	0.3	-	0.1

Marital status*								

Unmarried	-	33.1	-	35.6	-	8.7	-	23.9

Married	-	64.3	-	60.7	-	90.0	-	74.7

Separated	-	1.4	-	1.8	-	0.3	-	0.6

Divorced	-	0.4	-	1.0	-	0.1	-	0.1

Widowed	-	0.1	-	0.1	-	0.1	-	0.4

Missing	-	0.7	-	0.7	-	0.7	-	0.4

First prenatal visit (weeks)*								

<12	87.9	92.8	92.0	96.7	87.8	92.4	82.3	92.1

≥12	12.0	7.0	8.0	3.2	12.2	7.5	17.7	7.8

Missing	0.1	0.2	0.0	0.1	0.0	0.1	0.0	0.1

Parity*								

0	50.3	48.8	55.1	53.0	57.5	50.9	51.8	49.2

1	30.8	33.2	33.1	33.7	33.0	35.9	34.8	36.1

≥2	18.9	18.0	11.8	13.3	9.5	13.2	13.4	14.8

PERINATAL OUTCOMES								

Gestational age (weeks)								

<31	3.0	2.3	1.2	0.8	1.1	0.9	1.0	1.0

32-36	6.7	7.5	5.2	7.3	5.7	7.5	6.3	6.2

37+	88.6	90.2	92.5	91.9	91.8	91.6	90.7	92.8

Missing	1.7	0.0	1.2	0.0	1.4	0.0	1.9	0.0

Five Minute Apgar Score								

0-6	3.9	1.6	1.8	2.2	1.5	2.2	1.5	1.8

7-10	95.8	98.4	97.8	97.8	98.5	97.8	98.3	98.2

Missing	0.4	0.0	0.3	0.0	0.0	0.0	0.2	0.0

Respiratory diseases	11.4	7.3	7.9	4.9	7.2	5.5	8.0	4.5

Special/intensive care	8.0	7.7	4.5	4.9	5.0	5.9	6.4	3.8

Congenital Malformations	4.2	8.6	4.5	5.4	3.6	6.7	3.2	5.2

*Χ^2 ^test for differences in proportions statistically significant (p < 0.0001) across regions within each time period.

Percentages are by column.

### Outcomes

Table 2 shows the distribution of selected perinatal outcomes and table 3 shows the results from logistic regression models. Results from the multivariate analyses on mothers coming from DCs are not reported because their characteristics and risk patterns were similar to those observed among Italians.

**Table 3:** Mother's region of birth and perinatal outcomes: crude odds ratios (ORs) and robust 95% confidence intervals (95%CIs) from logistic regression models. Lazio, 1996-1998 and 2006-2008

Outcome	Model 1a: '96-'98		Model 1b: '06-'08		Model 2: '06-'08 vs. '96-'98	
	ORs	95%CIs	ORs	95%CIs	ORs	95%CIs
Gestational Age						
<31 vs.≥37						
Italy	1.00		1.00		0.88	(0.80-0.97)
Eastern Europe	1.65	(1.21-2.25)	2.11	(1.80-2.47)	1.12	(0.89-1.42)
North Africa	0.78	(0.56-1.10)	0.94	(0.46-1.93)	1.05	(0.40-2.76)
West and sub-Saharan Africa	4.55	(2.88-7.18)	3.91	(2.85-5.35)	0.76	(0.55-1.04)
Central and South America	1.71	(1.15-2.54)	1.29	(0.95-1.75)	0.66	(0.44-1.01)
Western and Central-Southern Asia	1.59	(0.80-3.17)	1.45	(0.86-2.45)	0.80	(0.46-1.39)
East Asia	1.54	(1.04-2.27)	1.71	(0.88-3.33)	0.98	(0.69-1.38)
ALL REGIONS	1.80	(1.44-2.28)	1.95	(1.72-2.21)	0.95	(0.77-1.16)
32-36 vs.≥37						
Italy	1.00		1.00		1.09	(1.05-1.13)
Eastern Europe	1.35	(1.06-1.72)	1.31	(1.19-1.46)	1.06	(0.87-1.30)
North Africa	1.48	(1.22-1.78)	0.98	(0.71-1.34)	0.72	(0.55-0.95)
West and sub-Saharan Africa	1.48	(1.11-1.97)	1.49	(1.27-1.75)	1.10	(0.89-1.37)
Central and South America	1.10	(0.92-1.31)	1.42	(1.26-1.62)	1.42	(1.12-1.79)
Western and Central-Southern Asia	1.21	(0.99-1.48)	1.47	(1.28-1.70)	1.32	(0.96-1.84)
East Asia	1.37	(0.92-2.04)	1.19	(0.92-1.57)	0.96	(0.80-1.14)
ALL REGIONS	1.32	(1.16-1.50)	1.32	(1.25-1.39)	1.09	(0.96-1.23)
Five Minute Apgar Score (0-6 vs. 7-10)						
Italy	1.00		1.00		0.81	(0.76-0.87)
Eastern Europe	1.13	(0.86-1.49)	1.02	(0.91-1.15)	0.73	(0.53-1.00)
North Africa	1.21	(0.80-1.82)	1.29	(0.93-1.79)	0.87	(0.72-1.04)
West and sub-Saharan Africa	2.30	(1.55-3.41)	1.17	(0.69-1.97)	0.41	(0.22-0.77)
Central and South America	1.07	(0.72-1.58)	1.57	(1.25-1.96)	1.19	(0.85-1.67)
Western and Central-Southern Asia	0.89	(0.49-1.61)	1.57	(0.90-2.74)	1.44	(0.55-3.72)
East Asia	0.88	(0.81-0.96)	1.32	(1.26-1.40)	1.22	(1.13-1.31)
ALL REGIONS	1.18	(0.99-1.40)	1.17	(1.03-1.34)	0.81	(0.66-1.00)
Respiratory diseases (yes vs. no)						
Italy	1.00		1.00		0.66	(0.63-0.68)
Eastern Europe	1.17	(1.10-1.24)	1.28	(1.22-1.34)	0.72	(0.67-0.77)
North Africa	1.31	(0.98-1.75)	1.40	(1.23-1.60)	0.70	(0.58-0.86)
West and sub-Saharan Africa	1.73	(1.46-2.05)	1.61	(1.34-1.93)	0.61	(0.50-0.75)
Central and South America	1.15	(0.98-1.35)	1.05	(0.94-1.18)	0.60	(0.51-0.71)
Western and Central-Southern Asia	1.05	(0.85-1.29)	1.20	(0.99-1.47)	0.75	(0.66-0.87)
East Asia	1.18	(1.03-1.35)	0.97	(0.72-1.29)	0.54	(0.44-0.65)
ALL REGIONS	1.22	(1.15-1.31)	1.24	(1.17-1.32)	0.66	(0.62-0.71)
Special/intensive care (yes vs. no)						
Italy	1.00		1.00		0.96	(0.92-1.00)
Eastern Europe	1.36	(1.22-1.52)	1.46	(1.33-1.59)	1.02	(0.93-1.13)
North Africa	1.29	(0.95-1.74)	1.28	(0.99-1.66)	0.95	(0.83-1.10)
West and sub-Saharan Africa	2.21	(1.73-2.82)	2.22	(1.89-2.60)	0.96	(0.76-1.21)
Central and South America	1.21	(0.97-1.50)	1.36	(1.16-1.60)	1.08	(0.91-1.28)
Western and Central-Southern Asia	1.35	(1.06-1.71)	1.66	(1.48-1.86)	1.18	(0.86-1.62)
East Asia	1.75	(1.41-2.16)	1.04	(0.65-1.66)	0.57	(0.43-0.77)
ALL REGIONS	1.47	(1.30-1.66)	1.45	(1.34-1.57)	0.95	(0.92-1.00)
Congenital malformations (yes vs. no)						
Italy	1.00		1.00		1.45	(1.40-1.51)
Eastern Europe	1.12	(0.96-1.30)	1.04	(0.98-1.10)	1.35	(1.13-1.60)
North Africa	0.84	(0.60-1.16)	1.12	(0.91-1.37)	1.94	(1.09-3.44)
West and sub-Saharan Africa	1.12	(0.89-1.39)	1.65	(1.45-1.86)	2.14	(1.69-2.72)
Central and South America	1.20	(0.94-1.53)	1.00	(0.90-1.11)	1.21	(0.93-1.57)
Western and Central-Southern Asia	0.94	(0.81-1.11)	1.25	(1.01-1.54)	1.92	(1.46-2.52)
East Asia	0.84	(0.66-1.08)	0.95	(0.83-1.09)	1.63	(1.40-1.91)
ALL REGIONS	1.03	(0.93-1.15)	1.07	(1.00-1.15)	1.50	(1.32-1.71)

Model 1a: effect of mother's region of birth (reference = Italians) on each outcome in 1996-1998.

Model 1b: effect of mother's region of birth (reference = Italians) on each outcome in 2006-2008.

Model 2: effect of time (reference = 1996-1998) on each outcome, stratified by mothers' region of birth. The group "ALL REGIONS" includes only immigrant mothers.

#### Gestational age

VPBs and PBs percentages were the highest among mothers from West and sub-Saharan Africa (7.5% and 2.3% in the second period, respectively).

Crude ORs of VPBs were higher among immigrants than among Italians both in the first (OR:1.80; 95%CI:1.44-2.28, model 1a) and the second time period (OR:1.95; 95%CI:1.72-2.21, model 1b), especially for mothers from West and sub-Saharan Africa (OR:4.55; 95%CI:2.88-7.18 in the first period, model 1a) and Eastern Europe (OR:2.11; 95%CI:1.80-2.47 in the second period, model 1b). The odds of VPBs decreased over time only among Italians (OR:0.88; 95%CI:0.80-0.97, model 2).

Similar results were observed for PBs, with mothers from West and sub-Saharan Africa again showing the highest odds. The odds of PBs actually increased over time (model 2) among Italians (OR:1.09; 95%CI:1.05-1.13) and among mothers from Central and South America (OR:1.42; 95%CI:1.12-1.79), while they decreased among North African mothers (OR:0.72; 95%CI:0.55-0.95).

#### Apgar score at 5 minutes

The percentage of receiving a low Apgar score was highest among West and sub-Saharan newborns in the first period (3.9%) and highest among North Africans in the second (1.8%).

Greater odds of a low Apgar reached statistical significance among immigrant mothers compared to Italian mothers only in the second period (OR:1.17; 95%CI:1.03-1.34, model 1b), in particular for those from Central and South America (OR:1.57; 95%CI:1.25-1.96, model 1b). Western and sub-Saharan Africans showed highest odds in the first period (OR:2.30;95%CI:1.55-3.41, model 1a). The odds was lower for East Asians in the first period (OR:0.88; 95%CI:0.81-0.96, model 1a) while it was higher in the second period (OR:1.32; 95%CI:1.26-1.40, model 1b) compared to Italians. The odds ratio of a low Apgar decreased over time (model 2) among Italians (OR:0.81; 95%CI:0.76-0.87) and mothers from West and sub-Saharan Africa (OR:0.41; 95%CI:0.22-0.77) while it increased among East Asians (OR:1.22; 95%CI:1.13-1.31).

#### Respiratory diseases

West and sub-Saharan Africans showed the highest percentages of respiratory diseases in both periods (11.4% and 7.3%, respectively), with crude odds of 73% (95%CI:1.46-2.05) and 61% (95%CI:1.34-1.93) higher than Italians, in the first and second period respectively (model1a and 1b). Respiratory diseases were more common among immigrants than in Italians especially in the second period (OR:1.24; 95%CI:1.17-1.32, model 1b), but the odds decreased over time among both groups (model 2).

#### Need for special or intensive neonatal care

A greater need for special or intensive neonatal care was observed among migrant origin infants compared to Italians. The highest percentages were observed among West and sub-Saharan Africans in both periods (8.0%, and 7.7%). They showed also the highest odds ratios (OR:2.22; 95%CI:1.89-2.60 in the second period, model 1b). The effect was higher among immigrants from all regions compared to Italians in particular in the first period (OR:1.47; 95%CI:1.30-1.66, model 1a). The odds decreased over time only among East Asians (OR:0.57; 95%CI:0.43-0.77, model 2).

#### Congenital malformations

The highest percentages of congenital malformations were observed in Central and South Americans in the first period (4.5%) and West and sub-Saharan Africans in the second (8.6%). The effect was higher among immigrants from all regions than among Italians in the second period (OR:1.07; 95%CI:1.00-1.15, model 1b), with highest odds ratios observed among sub-Saharan Africans (OR:1.65; 95%CI:1.45-1.86, model 1b). The odds of congenital malformations increased over time among all groups, in particular among West and sub-Saharan Africans (OR:2.14; 95%CI:1.69-2.72, model 2).

There was no interaction found between maternal birthplace and citizenship.

## Discussion

All perinatal adverse outcomes considered in our study occurred more commonly among infants of immigrants than Italians, independent of all confounders considered, except among mothers from developed countries that showed risk patterns similar to those observed among Italians. Being of Western or sub-Saharan African origin especially was positively associated with adverse perinatal outcomes. This finding may be due to a higher incidence of maternal diseases (e.g., hypertension, infectious diseases), and genetic differences [44]. In addition, social exclusion for cultural reasons, poor living conditions, less frequent and sub-optimal prenatal care may explain these results [2,6,14,45].

The recent increase in Romanian mothers, who had the lowest percentage of naturalised Italian citizens and therefore could be less integrated, and the presence of Roma people (about 7000 in Rome according to the Italian Red Cross in 2008) characterized by high birth rates and poor living conditions, may influence the results among Eastern Europeans. Suboptimal prenatal care due to a lack of information of women and poor training of health professionals [5] may also affect our results.

Over time, an overall increase in the odds was found only for late preterm live births and congenital malformations, but there were differences across regions, as found in Italian mothers.

Prematurity was associated with maternal immigrant status, especially among West and sub-Saharan Africans. This result may be explained by a higher incidence of maternal disease (e.g., pregnancy hypertension) or genetic differences [14,45]. It is possible that immigrant mothers with pregnancy-associated diseases received sub-optimal prenatal care leading to an unfavourable perinatal outcome. Similar findings were observed in a northern Italian city in which Africans were at greater risk of prematurity associated with short permanence in the country independent of timely access to prenatal care [12]. The higher prevalence rate of PBs among ethnic minority groups in a study conducted in Amsterdam was hypothetically due to earlier foetal maturation in black women [44]. Also a meta-analysis of the literature published from 1995 to 2008 showed a higher risk of PBs in Asians and Africans, and lower risk in Latin Americans [10].

The Apgar score at 5 minutes was lower among West and sub-Saharan Africans and central and southern Americans. This finding may be linked to prematurity, observed in particular among Africans, and to sub-optimal care during pregnancy. Similar results were found in Washington state among infants born to Somali women compared to US-born blacks and whites, with prolonged gestation as a possible explanation accounted by the authors [15]. In a Finnish study cited above the Apgar score at 1 minute was lowest among newborns of African- and Somali-origin; this result was attributed to variations in healthcare procedures during labour according to maternal origin [13]. The absence of differences in health status at birth between Eastern European and Italian-origin infants may be partially due to residual confounding of young maternal age at delivery of Eastern European mothers. Interestingly, poorer health at birth was observed among infants born to East Asian compared to Italian women in the second period: the recent increase of Chinese immigration, characterised by young mothers from low socioeconomic backgrounds, linguistic and cultural barriers that may limit access to prenatal care may explain this result. Comparison with other Italian settings is limited by the scarcity of studies on this outcome.

The odds of having respiratory disease were the highest among infants born to West and sub-Saharan African women. This result might be partially mediated by the prematurity observed among infants born to Africans. The result may be also linked to a lower use of prenatal steroids as a consequence of poor prenatal care. A similar result was found in a study carried out in several Italian neonatal centres where infants born to nomadic and African parents had a higher incidence of neonatal asphyxia compared to those of native-born parents [6]. Social disadvantage, difficult access to healthcare services, late or inadequate prenatal care were listed as possible explanations.

An association between maternal birth country and need for special or intensive neonatal care was observed, again particularly among West and sub-Saharan Africans. Very few studies have considered this outcome among immigrant mothers. In Spain a similar outcome was not more frequent among newborns of immigrant mothers compared to the host population [46]. In addition to poor perinatal health, this outcome may reflect the use of invasive procedures that in some cases may have an iatrogenic effect (e.g. retinopathy of prematurity [47]).

Congenital malformations were higher among immigrants compared to Italians, especially among West and sub-Saharan Africans. Few studies are published regarding the association between birth defects and migrant status, therefore there have not been many hypotheses proposed. An Italian study [7] found a higher rate of deformities among migrants compared to Italians possibly due to poor prenatal care. In another study it was argued that socio-economic and cultural factors may explain differences in congenital malformations between migrant and non-migrants [18]. In order to explain our result we hypothesised that these differences could be due to a higher occurrence of life threatening (anencephaly) or severe chromosomal anomalies (e.g., trisomy 13 or 18, Ebstein's anomaly) that could reflect less common use of legal termination of pregnancy among immigrant compared to Italian women. Point estimates confirmed this hypothesis (results not shown), although without statistical significance. Higher prevalence of risk factors for congenital malformations among immigrant mothers, such as lower periconceptional folic acid use, suboptimal control of preconceptional diabetes and epilepsy, obesity, maternal smoking, low vaccine coverage, higher alcohol consumption, and marriage between cousins in some migrant groups may also play a role. We cannot exclude that grouping birth defects may mask associations and that the different number of stillbirths in the two populations may indicate different prevalence of birth defects. Although the congenital anomalies data are not from a dedicated registry but from hospital records, we believe that any underestimate of birth defects made in the hospital environment was very unlikely.

The two time periods analysed allowed us to evaluate changes in the perinatal health in women of foreign origin. We observed a statistically significant decrease in the odds of very preterm live births only among Italian women; on the other hand, we found a slight increase of late preterm in both Italian and foreign women. This result may be in part explained by improved prenatal care in reducing the occurrence of very preterm births.

Improvement was also seen in decreases of other unfavourable outcomes (low Apgar score at 5 minutes, respiratory diseases, need for special or intensive neonatal care). The only worsening of perinatal outcomes regarded congenital malformations, which had about a fifty percent increase of odds ratio from the last to the first time period, in both groups. The high percentage of minor congenital anomalies observed may reflect an improvement in diagnostic assessment over the two periods in diagnostic assessment.

A limitation of our study is the lack of information in our administrative database of some risk factors such as direct measures of integration in the host country, socio-economic status, prenatal care, and life style (including tobacco use). However, we used proxy measures of acculturation (naturalized Italian citizens), socioeconomic status (educational level attained), and prenatal assistance (GA at the first visit). No reliable information was available on the fathers (e.g. country of origin, occupational status), which would have helped to interpret the results in relation to the families' social background and resources.

We could not analyse perinatal death as an outcome because our database does not list stillbirths or early neonatal death that occurred after transfer to another hospital. In most developed countries the etiology of perinatal death differs widely, and we expected this to be the case even more so when comparing immigrant and Italian birth experiences. For example, one might expect a higher prevalence of stillbirths among immigrant women if there are difficulties in prenatal access.

We could not identify illegal residents in the dataset. Since they may be those at greatest risk of unfavourable perinatal outcome, we cannot exclude outcome differences as a result.

It may be that some conditions may have been affected by changes in coding, hospital practices, or reporting over time. In addition, the association between maternal birthplace and outcomes may change over time if the composition of the region changes according to driving countries.

This study is one of the few to analyse the perinatal health status of immigrants in a wide region with significantly increasing immigration. Data were representative, as they covered all the births that occurred in the region; there were very low percentages of missing data except for GA (5% among Eastern European women). Out of hospital births were not available but this phenomenon is uncommon in our region (<0.1% in 2006) [48]. Availability of routine data over time allowed a temporal comparison. Given the likelihood of cultural, socio-economic, and integration differences between mothers coming from different countries, the opportunity to split data by area of origin enabled us to identify subgroups at high risk of negative perinatal outcomes.

## Conclusions

Our findings are consistent with those of most studies that have shown worse perinatal outcomes among immigrant mothers compared to the native-born population. Differences in perinatal outcomes between newborns of migrant-origin and Italians did not change over time, and overall adverse outcomes steadily decreased over time in both immigrant and Italian women. The improvement over time in perinatal outcomes of immigrant women may be explained by healthier immigrants now than a decade ago, and by policies adopted to increase the accessibility to mother-child health services during the periconception and prenatal periods for legal and illegal immigrant women in Italy.

## Competing interests

The authors declare that they have no competing interests.

## Funding

None.

## Authors' contributions

LC wrote the manuscript, was responsible for the statistical data analysis, interpreted the results. SA actively participated in writing the manuscript, performed the literature review, interpreted the results. AP suggested and performed the statistical analysis, participated in the interpretation of the results, revised the manuscript. FF suggested the statistical analysis, participated in the interpretation of the results, revised the manuscript. RL suggested the research questions, contributed to the literature review, interpreted the results, revised the manuscript suggesting important intellectual concepts. MDC suggested the research questions, contributed to the literature review, interpreted the results, revised the manuscript suggesting important intellectual concepts. DDL suggested the research questions and design of the study, participated in writing the manuscript, interpreted the results, revised the manuscript suggesting important intellectual concepts, supervised the research group. GG contributed to the conception of the study and revised the manuscript. All the authors have given final approval of the version to be published.

## Pre-publication history

The pre-publication history for this paper can be accessed here:

http://www.biomedcentral.com/1471-2458/11/294/prepub

## Supplementary Material

Additional file 1 Classification of countries. Click here for file
