# Supplementary material for: Perinatal outcomes among immigrant mothers over two periods in a region of central Italy
Source: BMC Public Health. 2011 May 10;11:294. doi: 10.1186/1471-2458-11-294 (PMC3119162; doi:10.1186/1471-2458-11-294)
Supplement: Additional file 1 — Classification of countries. [file 1471-2458-11-294-S1.DOC]

**Classification of countries**

**Western developed countries (DCs)**

Austria, Belgium, Canada, Cyprus, Denmark, Finland, France, Germany, Greece, Iceland, Ireland, Israel, Japan, Luxembourg, Malta, Monaco, Netherlands, New Zealand, Norway, Oceania, Portugal, Spain, Sweden, Switzerland, United Kingdom, United States.

**Eastern Europe**

Albania, Belarus, Bosnia-Herzegovina, Bulgaria, Croatia, Czech Republic, Estonia, Hungary, Latvia, Lithuania, Macedonia, Moldova, Poland, Romania, Russian Federation, Serbia-Montenegro, Slovakia, Slovenia, Turkey, Ukraine,

**North Africa**

Algeria, Egypt, Libyan Arab Jamahiriya, Morocco, Sudan, Tunisia

**West and sub-Saharan Africa**

Angola, Benin, Burkina Faso, Burundi, Cameroon, Cape Verde, Central African Republic, Chad, Congo Democratic Republic, Congo, Côte D'Ivoire, Djibouti, Equatorial Guinea, Eritrea, Ethiopia, Gabon, Gambia, Ghana, Guinea, Guinea-Bissau, Kenya, Lesotho, Liberia, Madagascar,Malawi, Mali, Mauritania, Mauritius, Mozambique, Namibia, Niger, Nigeria, Rwanda, Sao Tome and Principe, Senegal, Seychelles, Sierra Leone, Somalia, South Africa, Swaziland Tanzania United Republic, Togo, Uganda, Zambia, Zimbabwe.

**Central and South America**

Antigua and Barbuda, Argentina, Bahamas, Bolivia Plurinational State, Brazil, Chile, Colombia, Costa Rica, Cuba, Dominica, Dominican Republic, Ecuador, El Salvador, Guatemala, Haiti, Honduras, Jamaica, Mexico, Nicaragua, Panama, Paraguay, Peru, Saint Kitts and Nevis, Saint Vincent and The Grenadines, Suriname, Trinidad and Tobago, Uruguay, Venezuela.

**Western and Central-Southern Asia**

Afghanistan, Armenia, Azerbaijan, Bangladesh, Georgia, India, Iran Islamic Republic, Iraq, Jordan, Kazakhstan, Kuwait, Kyrgyzstan, Lebanon, Maldives, Nepal, Pakistan, Palestinian Territory, Saudi Arabia, Sri Lanka, Syrian Arab Republic, Tajikistan, United Arab Emirates, Uzbekistan, Yemen.

**East Asia**

Cambodia, China, East Timor, Indonesia, Korea Democratic People's Republic, Korea Republic, Lao People's Democratic Republic, Malaysia, Myanmar, Philippines, Singapore, Taiwan, Thailand, Vietnam.
